# The Effects of Temperature on Development, Reproduction, Population Dynamics and Protective Enzyme Activity of *Neotoxoptera formosana* (Hemiptera: Aphididae)

**DOI:** 10.3390/insects16030263

**Published:** 2025-03-03

**Authors:** Jiawei Liu, Yutong Feng, Weixian Yi, Changying Zheng, Lijuan Sun

**Affiliations:** 1 College of Plant Medicine, Shandong Engineering Research Center for Environmentally Friendly Agricultural Pest Management, Qingdao Agricultural University, Qingdao 266109, China; 15689947326@163.com (J.L.); 13064095141@163.com (Y.F.); 2 Huangdao Customs of the People’s Republic of China, Qingdao 266109, China; y3wx@163.com

**Keywords:** *Neotoxoptera formosana*, temperature, population parameters, protective enzyme activity

## Abstract

*Neotoxoptera formosana* is an allium vegetable pest that has increasingly threatened Chinese leek production in North China in recent years. The effects of temperature on the biological characteristics and physiology of *N. formosana* are not well understood. In this study, life tables were constructed at 12 °C, 16 °C, 20 °C, 24 °C, and 28 °C to explore the effects of temperature on the development, reproduction, and population dynamics of *N. formosana*, and to determine the threshold temperature (C) and the effective cumulative temperature (K) for each developmental stage of this aphid. The protective enzyme activity of *N. formosana* at three representative temperatures, 12 °C, 20 °C, and 28 °C, was determined to explore the physiological response of *N. formosana* to temperature. The result showed that the increasing temperature speeded up the development of *N. formosana*, but decreased the body size, and shortened the longevity and reproduction period of adults. The temperature exceeding 20 °C inhibited its reproduction. The *N. formosana* population was obviously inhibited by 28 °C with all the population parameters decreasing to be the lowest, especially the fecundity, which was 16.7 times lower than that at 20 °C. The population expanded the fastest at 20 °C, followed by 24 °C, 16 °C, and 12 °C, the slowest at 28 °C, with the population size merely increasing to 2.3 times at 60 days. The K values for the first, second, third, and fourth-instar nymphs were 1.852 °C, 5.029 °C, 4.747 °C and 5.979 °C, respectively, and the C values were 47.540 days °C, 30.817 days °C, 33.481 days °C and 32.294 days °C, respectively. The C value for nymphal aphids was 4.002 °C, and the K value was 145.252 days °C. The activity of superoxide dismutase and peroxidase of *N. formosana* at 28 °C was higher than at 12 °C and 20 °C, and the catalase activity of *N. formosana* was highest at 12 °C. In conclusion, the temperature had significant effects on population parameters and the physiological metabolism of *N. formosana*.

## Simple Summary

*Neotoxoptera formosana* is a pest that damages allium vegetables, which has caused increasing damage to Chinese leek production in recent years. Given that organic production of leeks avoids the use of chemical pesticides, we wished to explore the potential of using short-time high temperatures generated by closing the shed vent at noon to control leek aphids, but the effects of temperature on the biological characteristics and physiology of *N. formosana* are not well understood. In this study, the effects of temperature on development, reproduction, and population parameters were studied by constructing life tables. The threshold temperature and the effective cumulative temperature were calculated according to data from the life tables, and the effects of temperature on protective enzyme activity were determined in the laboratory. The findings from this study are helpful for understanding the thermal tolerance of *N. formosana* and providing reference for the temperature control of it in a greenhouse.

## 1. Introduction

*Neotoxoptera formosana* (Takahashi) is found throughout East Asia and was first discovered in Taiwan in 1921. This aphid has been shown to reproduce parthenogenetically. In the late 20th century, it was found to damage Welsh onions in various countries in South America such as Brazil and Argentina [1,2], and some European countries such as Netherlands, Germany and Italy, etc. [3,4]. In recent years, *N. formosana* has frequently occurred in China and caused great losses in the production of allium vegetables [5]. *N. formosana* was found to damage leeks through the whole growth period of leek, and the damage by *N. formosana* leads to yellowing of the leaf, drying and atrophy of the leaf tip, slow growth and dwarfing of plant, and even death of the roots [6]. *N. formosana* has been found to feed in the tillers or crevices between the basement blades of leeks and fakes thanatosis when disturbed, which causes difficulties in the control of this aphid. In addition to the harm caused by piercing and sucking, damage caused by the spread of allium virus diseases during the migration of winged aphids was also serious [2,7]. Sometimes, farmers are forced to destroy the affected leek beds and replant the crop [6,8]. In China, the problem of chemical residues in leeks is a concern to the whole of society, and therefore it is very important to explore nonchemical control measures in leek production.

Temperature is a key factor influencing aphid’s growth, development, survival, and reproduction [9]. When facing a drastic change in temperature, aphids often adjust their physiology to resist the temperature stress [10,11]. Aphids gain greater tolerance to heat or cold by shortening their life span and reducing population parameters [12,13]. Aphids also respond to temperature stress by changing ecological strategies. For example, *Schizaphis graminum* Rondani produces a large number of winged aphids to migrate away from adverse conditions when the external temperature increases rapidly [14]. Temperature regulates the differentiation of chromotype and the seasonal polymorphism of aphid progeny [15]. Temperature is also involved in the regulation of aphids’ behavior and flight ability [16,17]. The study of the effect of temperature on aphids is of great significance for aphid control in the climate change context.

The effect of temperature on aphids has been studied for many species, but the effect of temperature on *N. formosana* has not been reported, despite the fact that *N. formosana* frequently occurs in organic leek cultivation in China. Given that organic production of leeks avoids the use of chemical pesticides, we wished to explore non-chemical measures for the control of leek aphids. Studying the effects of temperature on populations of *N. formosana* is helpful in informing potential control measures for *N. formosana* by using high temperatures in the organic production of leeks. In order to clarify the effect of temperature on *N. formosana*, the response of the growth, reproduction, and population dynamics of *N. formosana* to temperature was studied by constructing age-specific life tables; the developmental threshold temperatures and effective cumulative temperature for different developmental stages were calculated based on the relation between development rate and temperature. The physiological response of *N. formosana* to temperature was estimated by measurement of protective enzyme activity under representative temperatures.

## 2. Materials and Methods

### 2.1. The Leeks

The host plant leeks *Allium tuberosum* (Xuejiu 791) were 1-year-old specimens and were planted in the experimental field of the Ecology Laboratory of Qingdao Agricultural University. Every 20th leek were transplanted into an 18 cm high (d = 9 cm) plastic pot and used for aphid culture in the laboratory. The leeks were cultivated in a mixture of loam soil and peat soil (purchased from ETEPEK, Latvia, Germany) with a volume ratio of 1:1, and water-soluble fertilizer deluted 1000 times was applied every 7 days (N:P_2_O_5_:K_2_O = 19:19:19, Haifa Industrial Chemistry Limited, Haifa, Israel).

### 2.2. Insects

*N. formosana* was collected from a laboratory colony at the Ecology Laboratory of Qingdao Agricultural University (Qingdao, China). These aphids were originally collected from the plantation base of Qingdao Happiness Hometown Organic Vegetable Co., Ltd. (120.528481° E, 36.637058° N, Qingdao, China) and were maintained in an artificial climate box (RDN-300, Ningbo Southeast Instrument Co., Ltd., Ningbo, China) at 20 °C, 16L:8D at a relative humidity of 60% ± 10% with the above-mentioned potted leeks as food, and the potted leek was replaced every two weeks to keep enough food for the aphid. The aphid used for the experiment was raised for more than 10 generations before the experiments. Adult aphids of similar body sizes were collected from the laboratory colony and were transferred into a Petri dish (d = 9 cm) with the bottom lined with a piece of filter paper; a leek with its root wrapped around by the moist paper for moisture retention was provided as food. The Petri dish was sealed with a plastic film that was punctured with a few very small holes to provide air and placed in an artificial climate box (RDN-300, Ningbo Southeast Instrument Co., Ltd., Ningbo, China), and the culture conditions were the same as that for population maintaining. The adults were removed 24 h after and the newborn aphids were collected for the experiment.

### 2.3. Life Table Construction

The leek used for food was treated according to the method for newborn aphid collection in Section 2.2 and put into the Petri dish mentioned above, and a newborn aphid was carefully transferred to the leek with a small brush. The aphids were cultured in the artificial climate box at 12 °C, 16 °C, 20 °C, 24 °C, and 28 °C, respectively, with an illumination intensity of 15,000 lx, a light–dark ratio of 16L:8D and relative humidity of 60 ± 10%. The treatment at 20 °C was the control. During the experiment, 1.5 mL of water-soluble fertilizer was injected into the rhizosphere of each leek every 3 days with a syringe to maintain growth. The *N. formosana* were observed every 24 h to record the molting, survival and emergence of adults. The newborn aphids were removed from the Petri dish every 24 h after the adult aphids began to breed, and the number of newborn aphids from each adult was recorded until the adult died. The body length and width of 1-day-old adult aphids were measured using a microscope with a micrometer. The length from the tip of the head to the end of the cauda was measured as the body length, and the maximum width abdomen was measured as the body width. Every 1-day-old adult aphid was transferred into a marked centrifugal tube of 200 ul and weighed with a 1/10,000 electronic scale (Sartoris, Germany) for every 5 tubes as one unit to obtain aphid weight. Sixty newborn nymphs were used to construct the life table for each temperature.

### 2.4. Assay of Protective Enzyme Activities

*N. formosana* was cultured at 12 °C, 20 °C, and 28 °C according to the method in Section 2.3, and 1-day-old adults were collected for enzyme activity determination. One hundred aphids were transferred into a pre-cooled glass grinder, and ground with pre-cooled 0.9% saline in an ice bath. The homogenate was transferred into a 2.0 mL centrifuge tube and centrifuged at 12,000 rpm in a cold centrifuge (4 °C) for 15 min. The supernatant was collected for determination of enzyme activity. The superoxide dismutase (SOD), catalase (CAT), and peroxidase (POD) activities were determined using kits (Nanjing Institute of Jiancheng Bioengineering, Nanjing, China), and the protein content in the samples was determined by the Coomassie Brilliant Blue staining method [18]. Three replicates were conducted for each treatment.

### 2.5. Statistical Analysis

The “Parthenogenetic reproduction mode” of the TWOSEX-MSChart software [19] was used to analyze the biological parameters of the *N. formosana* population. The mean biological parameters, including the developmental period, fecundity, intrinsic rate of increase (*r*), net reproductive rate (*R*_0_), mean generation period (*T*), finite rate of increase (*λ*), and the standard estimations were calculated using the bootstrap method with 100,000 runs [20]. The differences in the data were checked using a paired bootstrap test using the TWOSEX-MSChart software [19]. The TIMING-MSChart software was used to predict and analyze the population dynamics of *N. formosana* at different temperatures [21]. Excel 2010 software was used to calculate the developmental threshold temperature and effective cumulative temperature for each developmental stage of *N. formosana* and to process the data for body size, weight (every 20 aphids were treated as a replicate), and enzyme activity. The developmental threshold temperature and effective cumulative temperature were calculated using the least square method [22]. The Tukey LSD test of SPSS 27.0 (IBM, Armonk, NY, USA) was used to analyze the differences in body size and enzyme activity between different treatments.

## 3. Results

### 3.1. The Developmental Period of Nymphal Neotoxoptera formosana at Different Temperatures

As can be seen from Table 1, in the temperature range from 12 °C to 20 °C, the developmental period of each stage showed a shortening with the increase in temperature, and the developmental period for each stage differed between different temperatures (12 °C vs. 20 °C for first instar: *p* < 0.00001; 16 °C vs. 20 °C for first instar: *p* = 0.00888; 12 °C vs. 16 °C for first instar: *p* < 0.00001; 12 °C vs. 20 °C for second instar: *p* < 0.00001; 16 °C vs. 20 °C for second instar: *p* < 0.00001; 12 °C vs. 16 °C for second instar: *p* < 0.00001; 12 °C vs. 20 °C for third instar: *p* < 0.00001; 16 °C vs. 20 °C for third instar: *p<* 0.00001; 12 °C vs. 16 °C for third instar: *p* < 0.00001; 12 °C vs. 20 °C for fourth instar: *p* < 0.00001; 16 °C vs. 20 °C for fourth instar: *p* < 0.00001; 12 °C vs. 16 °C for fourth instar: *p* < 0.00001; 12 °C vs. 20 °C for nymphal stage: *p* < 0.00001; 16 °C vs. 20 °C for nymphal stage: *p* < 0.00001; 12 °C vs. 16 °C for fourth instar: *p* < 0.00001; 12 °C vs. 16 °C for nymphal stage: *p* < 0.00001). When the temperature was 24 °C, the speed of development decreased, and the developmental period for the second instar, third instar and fourth-instar nymph at 24 °C did not differ from those at 20 °C (second instar: *p* = 0.25979; third instar: *p* = 0.93838; and fourth instar: *p* = 0.13896). The most obvious slowing down of development was found at 28 °C; the developmental period for second-instar nymphs and third-instar nymphs did not differ from those at 20 °C (second-instar nymph: *p* = 0.90841; third-instar nymph: *p* = 0.17657), and the developmental period for fourth-instar nymphs was 0.5 days longer than at 20 °C.

### 3.2. The Biological Parameters of Adult Neotoxoptera formosana at Different Temperatures

As can be seen from Table 2, with the increase in temperature from 12 °C to 28 °C, an obvious shortening was found for the longevity and reproduction period of adult *N. formosana*. The longevity of adults at 12 °C was 28.8 days but merely 4.2 days at 28 °C, and the reproductive period of adults at 12 °C was 17.3 days but merely 2.3 days at 28 °C. The longevity of adult differing among all temperatures (12 °C vs. 20 °C for fecundity: *p* < 0.00001; 16 °C vs. 20 °C: *p* = 0.00003; 24 °C vs. 20 °C: *p* = 0.00030; 28 °C vs. 20 °C: *p* < 0.00001; 12 °C vs. 16 °C: *p* < 0.00001; 12 °C vs. 24 °C: *p* < 0.00001; 12 °C vs. 28 °C: *p* < 0.00001; 16 °C vs. 24 °C: *p* < 0.00001; 16 °C vs. 28 °C: *p* < 0.00001; 24 °C vs. 28 °C: *p* < 0.00001). The reproduction period of adults differed among all temperatures except between 12 °C and 16 °C (12 °C vs. 20 °C: *p* = 0.00136; 16 °C vs. 20 °C: *p* = 0.02587; 20 °C vs. 24 °C: *p* = 0.0029; 28 °C vs. 20 °C: *p* < 0.00001; 12 °C vs. 16 °C: *p* = 0.16089; 12 °C vs. 24 °C: *p* < 0.00001; 12 °C vs. 28 °C: *p* < 0.00001; 16 °C vs. 24 °C: *p* < 0.00001; 16 °C vs. 28 °C: *p* < 0.00001; 24 °C vs. 28 °C: *p* < 0.00001). The fecundity increased with the increase in temperature from 12 °C to 20 °C. The fecundity at 12 °C was 30.6 per female and differed from that at 20 °C (37.1 per female) (*p* = 0.04569). The fecundity at 24 °C was 23.7 per female and differed from that at 20 °C (*p* < 0.00001). The fecundity at 28 °C was merely 1.7 per female and differed from all the other treatments (28 °C vs. 12 °C: *p* < 0.00001; 28 °C vs. 16 °C: *p* < 0.00001; 28 °C vs. 20 °C: *p* < 0.00001; 28 °C vs. 24 °C: *p* < 0.00001).

### 3.3. The Population Parameters of Neotoxoptera formosana at Different Temperatures

As can be seen from Table 3, the mean generation time (*T*) of *N. formosana* decreased with the increase in temperature and differed between different temperatures (28 °C vs. 12 °C: *p* < 0.00001; 28 °C vs. 16 °C: *p* < 0.00001; 28 °C vs. 20 °C: *p* = 0.01676; 28 °C vs. 24 °C: *p* = 0.01745; 16 °C vs. 12 °C: *p* < 0.00001; 20 °C vs. 12 °C: *p* < 0.00001; 24 °C vs. 12 °C: *p*< 0.00001; 20 °C vs. 24 °C: *p* = 0.02781; 16 °C vs. 20 °C: *p* < 0.00001; 16 °C vs. 24 °C: *p* < 0.00001). When the treatment temperature increased from 12 °C to 20 °C, the intrinsic rate (*r*) of increase and the finite rate of increase (*λ*) of the population increased significantly and differed between temperatures (12 °C vs. 20 °C: *p* < 0.00001; 16 °C vs. 20 °C: *p* < 0.00001; 12 °C vs. 16 °C: *p* < 0.00001). The net reproductive rate (*R*_0_) of the *N. formosana* population was highest at 20 °C and differed from that at all other temperatures except 16 °C (20 °C vs. 12 °C: *p* = 0.00192; 20 °C vs. 16 °C: *p* = 0.52626; 20 °C vs. 24 °C: *p* < 0.00001; 20 °C vs. 28 °C: *p* < 0.00001). When the treatment temperature was 24 °C or 28 °C, all the population parameters decreased and each population parameter was lowest at 28 °C. The *R*_0_ at 28 °C was 1.18 and differed from that at other temperatures (28 °C vs. 12 °C: *p* < 0.00001; 28 °C vs. 16 °C: *p* < 0.00001; 28 °C vs. 20 °C: *p* < 0.00001; 24 °C vs. 28 °C for *R*_0_: *p* < 0.00001). The *T* at 28 °C was 11.60 and differed from that at other temperatures (28 °C vs. 12 °C: *p* < 0.00001; 28 °C vs. 16 °C: *p* < 0.00001; 28 °C vs. 20 °C: *p* = 0.01676; 24 °C vs. 28 °C: *p =* 0.01745). The *r* at 28 °C was 0.014 d^−1^ and differed from that at other temperatures (28 °C vs. 12 °C: *p* = 0.00567; 28 °C vs. 16 °C: *p* = 0.00035; 28 °C vs. 20 °C: *p* = 0.00001; 28 °C vs. 24 °C: *p* = 0.00007). The *λ* at 28 °C was 1.014 d^−1^ and differed from that at other temperatures (28 °C vs. 12 °C: *p* = 0.00359; 28 °C vs. 16 °C: *p* = 0.00006; 28 °C vs. 20 °C: *p* < 0.00001; 28 °C vs. 24 °C: *p* = 0.00001).

### 3.4. Age-Instar Survival Rate of Neotoxoptera formosana at Different Temperatures

**Figure 1:**
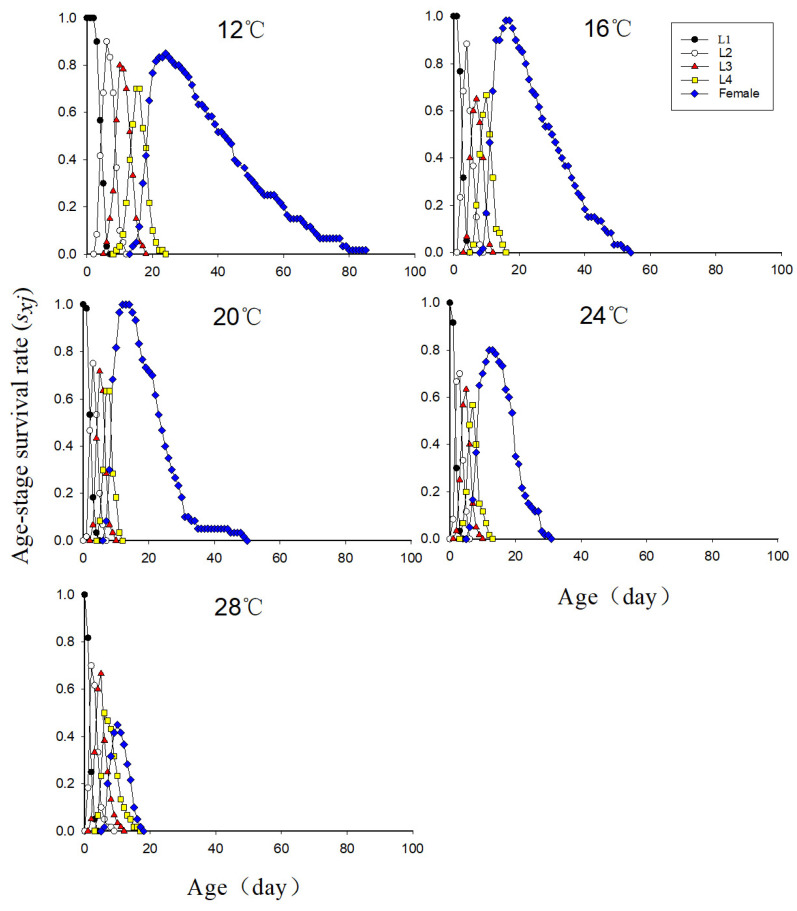
Age-stage survival rate (*s_xj_*) of *Neotoxoptera formosana* at different temperatures.

As can be seen from Figure 1, the preadult survival rate of *N. formosana* at 24 °C was obviously lower than those at 16 °C and 20 °C (24 °C vs. 16 °C, *p* = 0.0007; 24 °C vs. 20 °C: *p* = 0.0007). The preadult survival rate of *N. formosana* was lowest at 28 °C, and differed from that at other temperatures except 24 °C (28 °C vs. 12 °C: *p* = 0.0142; 28 °C vs. 16 °C: *p* < 0.0001; 28 °C vs. 20 °C: *p* < 0.0001; 28 °C vs. 24 °C: *p* = 0.0885). The high temperature exerted the most obvious effect on the survival of old nymphal aphids.

### 3.5. The Age-Specific Survival Rate and Fecundity of the Neotoxoptera formosana Population at Different Temperatures

**Figure 2:**
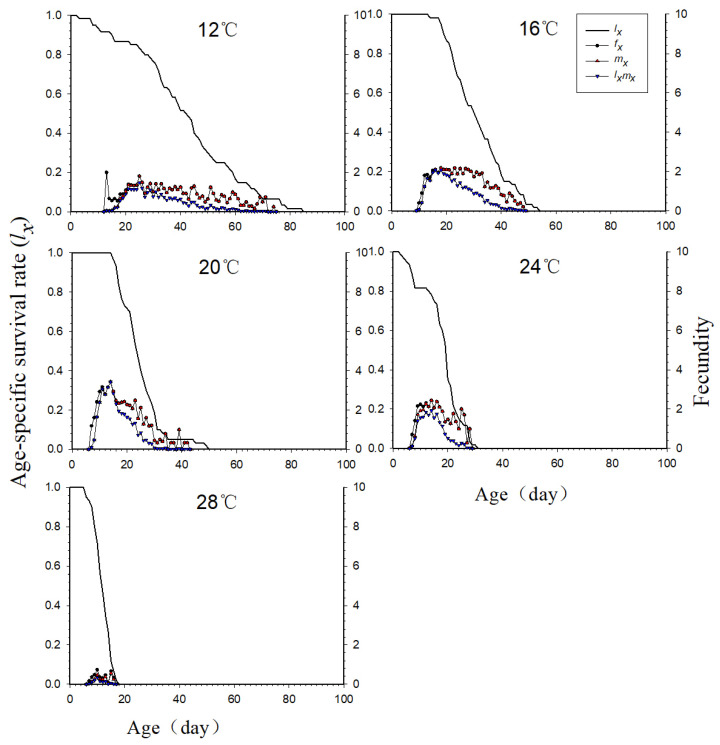
Age-specific survival rate (*l_x_*), age-stage specific fecundity (*f_x_*), age-specific fecundity (*m_x_*), and age-specific maternity (*l_x_m_x_*) of *Neotoxoptera formosana* at different temperatures.

As can be seen from Figure 2, the fertility of female adults (*f_x_*), population fertility (*m_x_*), and net fecundity of the population (*l_x_m_x_*) were highest at 20 °C and lowest at 28 °C. The turning point for the above parameters changing from high to low was 24 °C. The reproduction period of adult *N. formosana* was different at different temperatures. Adult *N. formosana* gave birth to progeny on the 13th day at 12 °C and ended the reproductive period on the 76th day; the reproductive period lasted for 63 days. The reproductive period of adult *N. formosana* at 24 °C was shorter than that at 16 °C and 20 °C (24 °C vs. 16 °C: *p* < 0.00001; 24 °C vs. 20 °C: *p* = 0.00290). The reproduction periods of adults were shortest at 28 °C and only lasted for 12 days and differed from that at other temperatures (*p* < 0.00001).

### 3.6. Effects of Different Temperatures on Life Expectancy of Neotoxoptera formosana

**Figure 3:**
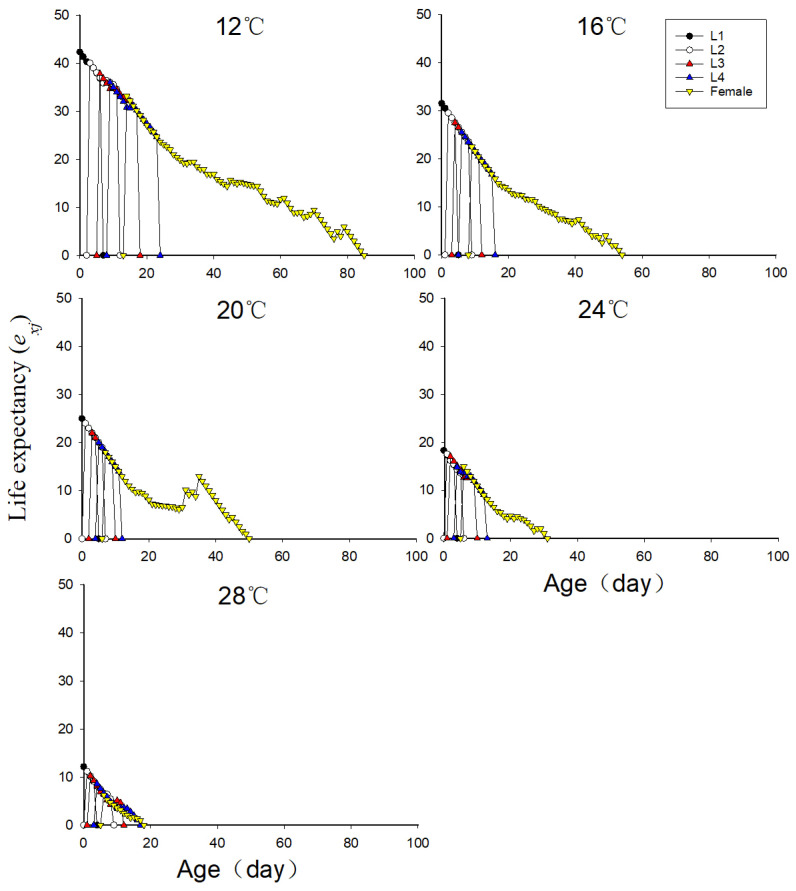
Age-stage specific life expectancy (*e_xj_*) of *Neotoxoptera formosana* at different temperatures.

As can be seen from Figure 3, the life expectancies of adult *N. formosana* decreased with the increase in temperature. The life expectancy of adult *N. formosana* was highest at 12 °C, which was 85 days. The life expectancy at 16 °C, 20 °C, and 24 °C was 54 days, 50 days, and 31 days, respectively. The life expectancy was lowest at 28 °C at 18 days. The high temperature exerted the most obvious effect on the life expectancy of the first-instar nymph.

### 3.7. Effects of Different Temperatures on the Population Reproduction Value of Neotoxoptera formosana

**Figure 4:**
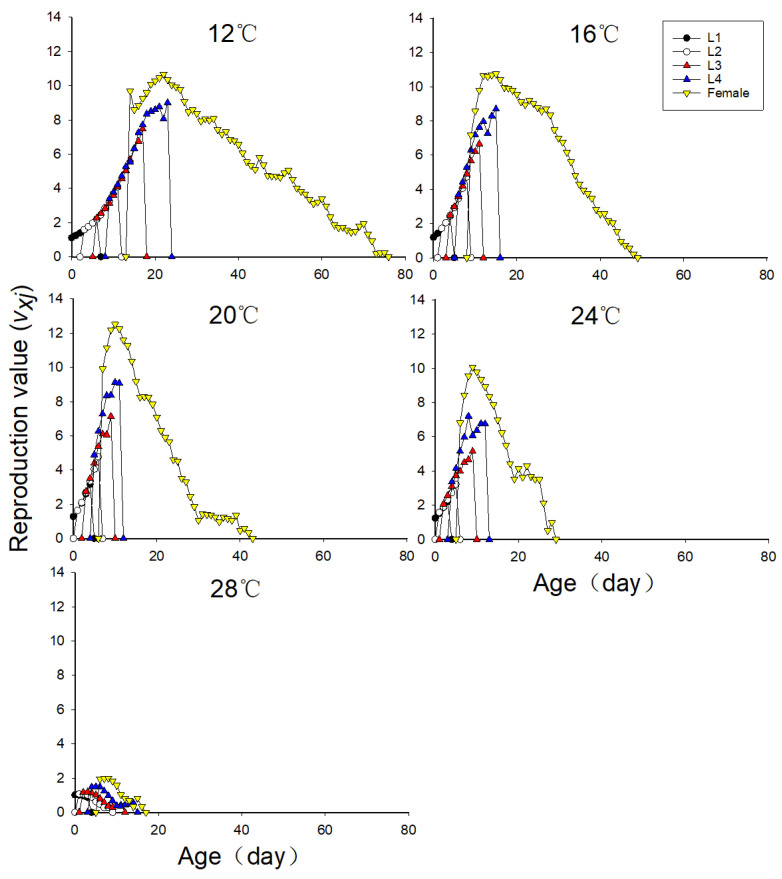
Age-stage reproductive values (*v_xj_*) of *Neotoxoptera formosana* at different temperatures.

As can be seen from Figure 4, the maximum reproductive value and occurrence of the reproductive peak were both affected by temperature. The maximum reproductive values increased with the increase in temperature and were 10.7 per female, 11.0 per female and 12.5 per female at 12 °C, 16 °C and 20 °C, respectively. At 24 °C, the maximum reproductive value declined to 10.1 per female. The maximum reproductive value was the lowest at 28 °C: 2.0 per female. The reproductive peak at 12 °C, 16 °C, 20 °C, 24 °C, and 28 °C occurred on the 26th day, 16th day, 10th day, 9th day, and 7th day, respectively; the higher the temperature, the earlier the reproductive peak occurs. The high temperature exerted the most obvious effect on the reproduction value of adult aphids.

### 3.8. The Predicted Population Trends of Neotoxoptera formosana at Different Temperatures

**Figure 5:**
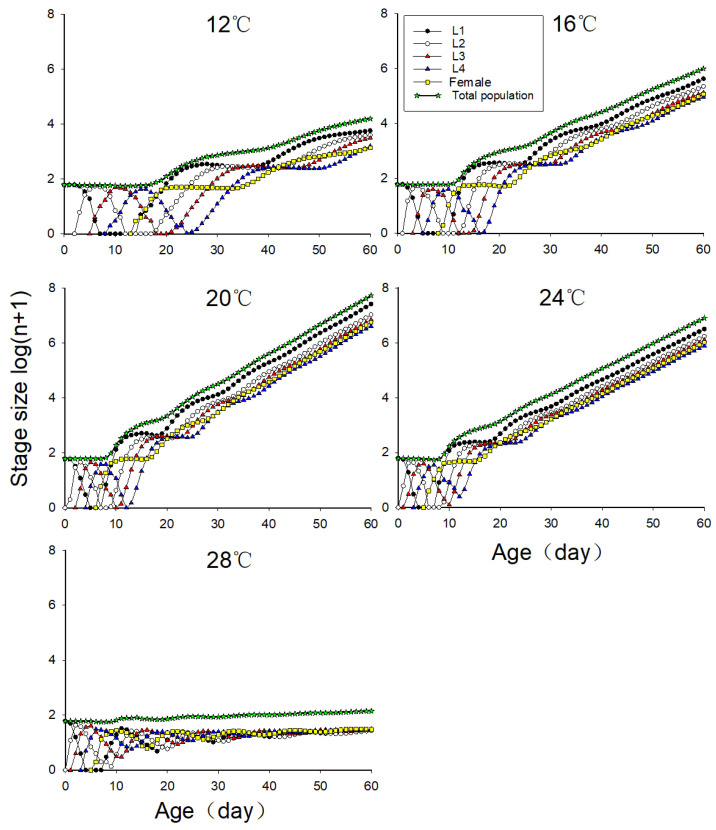
The population growth and decline trend of *Neotoxoptera formosana* at different temperatures.

As can be seen from Figure 5, the growth rate of the *N. formosana* population dramatically increased with the increase in temperature from 12 °C to 20 °C. On the 60th day, the population size at 12 °C, 16 °C, and 20 °C had increased by 238.4, 1.7 × 10^4^, and 9.1 × 10^5^ times under ideal food conditions, respectively. The population growth slowed down at 24 °C, and the population size increased by 1.3 × 10^5^ times under ideal food conditions. When the temperature was 28 °C, the population size only increased by 2.3 times under ideal food conditions.

### 3.9. Body Size and Weight of Neotoxoptera formosana at Different Temperatures

As can be seen from Table 4, the body size of adult *N. formosana* significantly decreased with the increase in temperature. The body size (length × width) for adult aphids at 12 °C was 1.722 mm × 0.958 mm, but that at 28 °C was only 1.303 mm × 0.700 mm; the body length and body width differed at different temperatures (body length: *df*_1_ = 4, *df*_2_ = 10, F = 925.3003; *p* <0.001; body width: *df*_1_ = 4, *df*_2_ = 10, F = 1039.8161; *p* < 0.001). The weight of adult aphids also significantly decreased with the increase in temperature, from 0.51 mg at 12 °C to 0.21 mg at 28 °C, and differed between different temperatures (*df*_1_ = 4, *df*_2_ = 10, F = 44.3334; *p* < 0.001).

### 3.10. Developmental Starting Point Temperature and Effective Cumulative Temperature of Neotoxoptera formosana

It can be seen from Table 5 that the developmental threshold temperature for first-instar nymphs, second-instar nymphs, third-instar nymphs, and fourth-instar nymphs was 1.85 °C, 5.03 °C, 4.75 °C, 5.03 °C, and 5.03 °C, respectively, and that for the whole nymph stage, it was 4.00 °C. The effective cumulative temperatures for first-instar nymphs, second-instar nymphs, third-instar nymphs, and fourth-instar nymphs were 47.5 days °C, 30.8 days °C, 33.5 days °C and 32.3 days °C, respectively, and that for the whole nymph stage it was 145.3 days °C.

### 3.11. Protective Enzyme Activity of Neotoxoptera formosana at Different Temperatures

**Figure 6:**
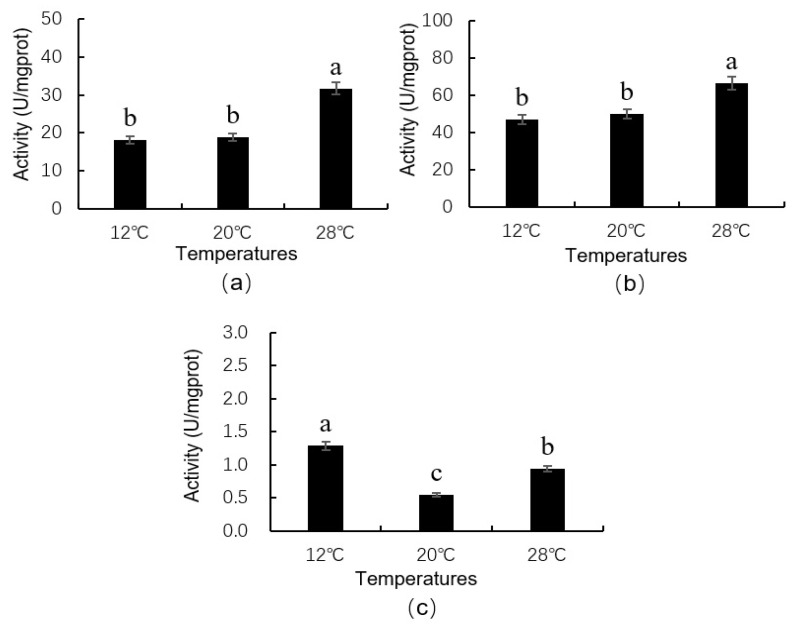
Activities of three protective enzymes of *Neotoxoptera formosana* at different temperatures. (**a**), (**b**) and (**c**) was the activity for POD, SOD and CAT, respectively. Data in the figure represent the mean ± SD. Different lowercase letters on the columns indicate a significant difference between enzyme activities at different temperatures at *p* < 0.05 according to LSD by one-way ANOVA using SPSS 27.0.

As can be seen from Figure 6, temperature exerted an obvious influence on the activities of protective enzymes of *N. formosana*. POD activity was highest at 28 °C (31.66 U/mgprot) (Figure 6a), and differed from that at 12 °C or 20 °C (*df*_1_ = 2, *df*_2_ = 6, F = 32.7616, *p* = 0.0006). SOD activity was also highest at 28 °C (66.68 U/mgprot), and differed from that at 12 °C or 20 °C (*df*_1_ = 2, *df*_2_ = 6, F =13.3172, *p* = 0.0062). The activities of POD and SOD did not differ between 12 °C and 20 °C (Figure 6b). The response of CAT enzyme activity to temperature was different from that of the above two enzymes. The highest CAT activity was found at 12 °C (1.29 U/mgprot), followed by that at 28 °C (0.94 U/mgprot), and the lowest activity was found at 20 °C (0.55 U/mgprot) (Figure 6c); the activity of CAT differed significantly among different temperatures (*df*_1_ = 2, *df*_2_ = 6, F = 17.930, *p* = 0.0029).

## 4. Discussion

Temperature exerted effects on several population parameters of *N. formosana* in this study, and the effects of temperature on development and reproduction were most obvious; the increasing temperature promoted its development, but the temperature higher than 24 °C inhibited its reproduction. Seo et al. [23] found that *Aulacorthum solani* could survive at temperatures between 5 °C and 27.5 °C, but the net reproduction rate was highest at 20 °C, and the higher temperature was not beneficial to reproduction. Similar results were obtained in research on *Semiaphis heraclei* (Takahashi) [24] and *Trichosiphonaphis lonicerae* Uye [25]. In this study, we found that *N. formosana* completed its life history at all the tested temperatures from 12 °C to 28 °C. However, the treatment temperature of 28 °C not only suppressed the reproduction of adults, but also shortened their longevity, and therefore population parameters including the net reproductive rate, the mean generation time, the intrinsic rate of increase, and the finite rate of increase decreased significantly. This finding provided further evidence for the adverse effect of high temperatures on aphids.

Using high temperatures to control pest insects in production facilities is an environmentally friendly control measure. According to Gu et al. [26], high temperatures in closed sheds significantly inhibited the occurrence of *Bemisia tabaci* (Gennadius). Yu et al. [27] found that the control rate of *Meggalurothrips usitatus* (Bagnall) was more than 70% at high temperatures in closed sheds, and this was also found effective in the control of tomato aphids [28], cucumber aphids [29] and *Bradysia odoriphaga* Yang et Zhang [30]. In this study, it was found that 28 °C significantly inhibited the reproduction of *N. formosana*, suggesting that it may be possible to use high temperatures in closed sheds to control this aphid. All insects, including aphids, exhibit varying degrees of thermal plasticity under high-temperature stress, which poses a challenge to using high temperatures to prevent pests [31]. The feasibility of using high temperatures to control *N. formosana* depends on further study on the effect of temperature (including constant temperature and fluctuating temperature) on its progeny.

In this study, 12 °C to 20 °C was the most appropriate range for the development and reproduction of *N. formosana*, so the developmental threshold temperature and the effective cumulative temperature were calculated according to the data for 12 °C, 16 °C, and 20 °C. It was found that the developmental threshold temperature of the whole nymph stage of *N. formosana* was 4.002 °C and that *N. formosana* developed well at 12 °C; these phenomena indicate that lower temperatures are favored by this aphid. Therefore, farmers must be vigilant in checking for this aphid in field cultivation of leeks in spring and autumn, as well as in greenhouse cultivation of leeks in winter.

SOD, CAT and POD are important protective enzymes in insects, which have the functions of scavenging free radicals, anti-oxidation, and protecting cells from internal harmful factors [32]. Stress caused by environmental factors such as temperature usually leads to changes in the activity of protective enzymes [33]. In this study, the POD and SOD enzyme activities of *N. formosa* cultured at high temperatures were higher than those at a low or moderate temperature; this result coincides with the studies of *Chilo suppressalis* Walker [34], *Antheraea mylitta* Drury [35], *B. tabaci* [36], *Propylaea japonica* (Thunberg) [37], and *Dysmicoccus neobrevipes* Beardsley [38]. CAT, like POD, plays an important role in the breakdown of H_2_O_2_ in insects [39]. In various studies, it was found that the same stress did not cause consistent changes in POD and CAT [36]. The same phenomenon was found in our study. Under the condition of 28 °C, POD activity was higher but CAT activity was lower, indicating that different protective enzymes have their own adaptable temperature ranges [36]. This result on the activities of three protective enzymes activities of *N. formosana* at different temperatures explained the mechanism of high temperature inhibiting this aphid from the perspective of enzyme activity.

Zhang et al. [40] explored the mechanism by which high temperatures suppress aphid populations from the aspect of nutrient metabolism. They found that high temperatures tended to cause excessive nutrient consumption by insects, resulting in smaller body size, delayed development, and decreased fertility. An adverse effect of high temperature on body size, weight, and fertility was also found for *N. formosana* in our study; therefore, the mechanism by which high temperatures may suppress nutrient metabolism in this aphid needs further study. Furthermore, we think the effect of temperature on *N. formosana* is not only a direct one but also an indirect one mediated by the host plant. The change in temperature may change the growth and material composition (including secondary compounds) of leeks and therefore influence the physiological metabolism of *N. formosana*, for example, the change in protective enzyme activity. The extent to which the temperature exerted on aphids was mediated by host plants needs to be evaluated in future studies. Chen et al. [41] reported temperature affected the abundance of aphid symbionts which was essential for the development and survival of aphids; therefore, the role of endosymbionts for thermotolerance or cold tolerance of aphids deserves further investigation.

## Figures and Tables

**Table 1:** Developmental period of *Neotoxoptera formosana* at different temperatures.

Developmental Stage	12 °C	16 °C	20 °C	24 °C	28 °C
N_1_	4.8 ± 0.1 a	3.1 ± 0.1 b	2.7 ± 0.1 c	2.3 ± 0.1 d	2.1 ± 0.1 d
N_2_	4.2 ± 0.2 a	3.0 ± 0.1 b	2.0 ± 0.1 c	1.9 ± 0.1 c	2.0 ± 0.1 c
N_3_	4.7 ± 0.2 a	2.9 ± 0.1 b	2.2 ± 0.1 c	2.2 ± 0.1 c	2.5 ± 0.2 c
N_4_	4.6 ± 0.2 a	2.9 ± 0.1 b	2.2 ± 0.1 d	2.4 ± 0.1 dc	2.7 ± 0.2 c
N	18.4 ± 0.3 a	11.9 ± 0.2 b	9.2 ± 0.2 c	8.7 ± 0.2 d	9.1 ± 0.4 c

Note: N_1_, N_2_, N_3_, N_4_, and N in the table represent 1st instar nymph, 2nd instar nymph, 3rd instar nymph, 4th instar nymph, and the whole nymphal stage. The data in the table are expressed as mean ± SE, and the standard errors were estimated by 100,000 bootstrap resamples. The lowercase letters after the data indicate significant differences between different treatments based on the paired bootstrap test at *p* < 0.05 by TWOSEX-MSChart software, 2024. The same applies below.

**Table 2:** Biological parameters of adult *Neotoxoptera formosana* at different temperatures.

Parameters	12 °C	16 °C	20 °C	24 °C	28 °C
*L* (d)	28.8 ± 2.1 a	19.7 ± 1.3 b	15.9 ± 1.0 c	12.2 ± 0.4 d	4.2 ± 0.4 e
*RP* (d)	17.3 ± 1.3 a	14.9 ± 1.0 a	12.3 ± 0.6 c	9.7 ± 0.6 d	2.3 ± 0.4 e
*F*	30.6 ± 2.7 b	35.1 ± 2.5 a	37.1 ± 2.0 a	23.7 ± 2.0 c	1.7 ± 0.5 d

Note: *L*, *RP* and *F* in the table represent the longevity of adults, the reproductive period of adults and average fecundity per female, respectively.

**Table 3:** The population parameters of *Neotoxoptera formosana* at different temperatures.

Parameters	12 °C	16 °C	20 °C	24 °C	28 °C
*R*_0_	26.60 ± 2.71 b	35.10 ± 2.51 a	37.15 ± 2.01 a	19.38 ± 2.03 c	1.18 ± 0.37 d
*T* (d)	29.60 ± 0.56 a	19.75 ± 0.36 b	14.68 ± 0.25 c	13.95 ± 0.21 d	11.60 ± 0.88 e
*r* (d^−1^)	0.110 ± 0.003 d	0.180 ± 0.003 c	0.246 ± 0.004 a	0.212 ± 0.008 b	0.014 ± 0.030 e
*λ* (d^−1^)	1.117 ± 0.003 d	1.197 ± 0.003 c	1.279 ± 0.005 a	1.236 ± 0.009 b	1.014 ± 0.030 e

Note: *R*_0_, *T*, *r*, *λ* in the table represent the net reproductive rate, the mean generation time, the intrinsic rate of increase, and the finite rate of increase, respectively.

**Table 4:** The body size and weight of adult *Neotoxoptera formosana* at different temperatures.

Parameters	12 °C	16 °C	20 °C	24 °C	28 °C
Body length (mm)	1.772 ± 0.009 a	1.564 ± 0.004 b	1.464 ± 0.004 c	1.413 ± 0.004 d	1.303 ± 0.005 e
Body width (mm)	0.958 ± 0.004 a	0.812 ± 0.001 b	0.763 ± 0.005 c	0.746 ± 0.002 d	0.700 ± 0.002 e
Weight (mg)	0.51 ± 0.01 a	0.42 ± 0.02 b	0.30 ± 0.03 c	0.25 ± 0.01 cd	0.21 ± 0.02 d

Note: Data in the table represent the mean ± SD. Different lowercase letters in the same line indicate a significant difference between data at different temperatures according to LSD at *p* < 0.05 by one-way ANOVA using SPSS 27.0.

**Table 5:** The developmental threshold temperature and effective cumulative temperature of *Neotoxoptera formosana.*

Stages	Developmental Threshold Temperature (°C)	Effective Cumulative Temperature (Day °C)	Formula for Development Rate
N_1_	1.850 ± 0.023	47.540 ± 3.577	V = (T − 1.851)/47.540
N_2_	5.030 ± 0.025	30.817 ± 3.545	V = (T − 5.029)/30.817
N_3_	4.747 ± 0.007	33.481 ± 3.019	V = (T − 4.747)/33.481
N_4_	4.979 ± 0.001	32.294 ± 0.007	V = (T − 4.979)/32.294
N	4.002 ± 0.003	145.252 ± 7.193	V = (T − 4.001)/145.252

Note: N_1_, N_2_, N_3_, N_4_, and N in the figure represent 1st instar nymph, 2nd instar nymph, 3rd instar nymph, 4th instar nymph, and the whole nymphal stage. The data for developmental threshold temperature and the effective cumulative temperature in the table were expressed as mean ± SD, and SD was calculated according to the least square method.

## Data Availability

The data presented in this study are available upon request from the corresponding author.
